# Diagnostic performance of GcfDNA in kidney allograft rejection: a meta-analysis

**DOI:** 10.3389/fphys.2023.1293402

**Published:** 2024-01-09

**Authors:** Hongji Yang, Duo Wang, Xin Sun, Hailian Wang, Yang Lan, Liang Wei

**Affiliations:** ^1^ Clinical Immunology Translational Medicine Key Laboratory of Sichuan Province, Sichuan Provincial People’s Hospital, School of Medicine, University of Electronic Science and Technology of China, Chengdu, China; ^2^ Transplantation Center, Sichuan Provincial People’s Hospital, School of Medicine, University of Electronic Science and Technology of China, Chengdu, China; ^3^ Chinese Evidence-Based Medicine Center and Chinese Cochrane Center, West China Hospital, Sichuan University, Chengdu, China

**Keywords:** graft-derived cell-free DNA, kidney transplantation, diagnostic performance, rejection, meta-analysis

## Abstract

In this comprehensive meta-analysis, our objective was to evaluate the diagnostic utility of graft-derived cell-free DNA (GcfDNA) in kidney allograft rejection and explore associated factors. We conducted a thorough search of PubMed, Embase, and the Cochrane Library databases, spanning from their inception to September 2022. Statistical analysis was executed utilizing Stata 15, Meta-DiSc 1.4, and Review Manager 5.4 software. The combined pooled sensitivity, specificity, positive likelihood ratio (PLR), negative likelihood ratio (NLR), diagnostic odds ratio (DOR), and the area under the summary receiver operating characteristics (SROC) curve from the synthesis of findings across ten studies were as follows: 0.75 (0.67–0.81), 0.78 (0.72–0.83), 3.36 (2.89–4.35), 0.32 (0.24–0.44), 8.77 (4.34–17.74), and 0.83 (0.80–0.86), respectively. Among the ten studies primarily focused on GcfDNA’s diagnostic potential for antibody-mediated rejection (ABMR), the optimal cut-off threshold demonstrated substantial diagnostic efficacy, with pooled sensitivity, specificity, positive likelihood ratio, negative likelihood ratio, DOR, and area under the summary receiver operating characteristics curve values of 0.83 (0.74–0.89), 0.75 (0.70–0.80), 3.37 (2.64–4.30), 0.23 (0.15–0.36), 14.65 (7.94–27.03), and 0.85 (0.82–0.88), respectively. These results underscore the high diagnostic accuracy of GcfDNA in detecting rejection. Furthermore, the optimal cut-off threshold proves effective in diagnosing ABMR, while a 1% threshold remains a robust diagnostic criterion for rejection. Notably, for ABMR diagnosis, droplet digital PCR digital droplet polymerase chain reaction emerges as a superior method in terms of accuracy when compared to other techniques. Nonetheless, further research is warranted to substantiate these findings.

## Introduction

Kidney transplantation stands as the most effective remedy for individuals afflicted by end-stage kidney disease. Despite significant advancements in graft survival, allograft rejection persists as a formidable challenge. Notably, in United States and numerous other nations, acute rejection within the first year post-kidney transplantation occurs in approximately 12% of cases (McDonald, 2015; Hart et al., 2021). Among adult recipients, the incidence rate of acute kidney allograft rejection (AR) hovers around 7.8% (Wolfe et al., 1999; Hart et al., 2019). Thus, early diagnosis holds pivotal clinical significance. However, contemporary methods for monitoring allograft injury, including markers such as serum creatinine, urinary protein, urinalysis, donor-specific antibodies, and BK virus surveillance, are encumbered by limitations in sensitivity and specificity (Schinstock et al., 2017; Cheungpasitporn et al., 2018; Leeaphorn et al., 2020). Serum creatinine, although a sensitive marker for evaluating glomerular filtration rate (GFR), lacks the requisite sensitivity and specificity for diagnosing allograft rejection, and monitoring its trends offers meager predictive value for detecting active rejection. Kidney needle biopsy, the gold standard for diagnosing rejection, proves unsuitable for frequent monitoring owing to its invasive nature and potential complications, such as gross hematuria and hematoma (Schwarz et al., 2005; Redfield et al., 2016). It was reported that up to 25% of biopsies yield an inadequate specimen (Oellerich et al., 2021). A study has suggested that performing protocol biopsies yields no significant benefits in terms of rejection rates, graft survival, or kidney function within the first 12 months post-transplant (Redfield et al., 2016). Due to complications, sampling error and variability in the interpretation of histological findings, rejection reactions with negative biopsy results may occur. One study showed “biopsynegative” rejection occurs in up to 20% of patients (Miller et al., 2013; Moein et al., 2023). Therefore, the quest for noninvasive diagnostic biomarkers boasting high sensitivity and specificity to facilitate optimal management of kidney transplant patients is of paramount importance.

Allograft transplantation exhibits unique allogenic genomic characteristics and can be conceived of as genomic transplantation. The investigation into plasma donor DNA as a potential biomarker of rejection dates back to 1998. Graft-derived cell-free DNA (GcfDNA), emanating from the necrotic or apoptotic cells of transplanted organs, emerges as a potential universal noninvasive biomarker for assessing allograft health (Lo et al., 1998). During periods of stable graft function, GcfDNA circulates at low levels. However, in cases of injury, including rejection, significantly elevated levels of GcfDNA are released into the bloodstream, signifying organ damage in solid organ transplantation. The quantification of GcfDNA can be accomplished through next-generation sequencing (NGS) or droplet digital PCR (ddPCR) and can be expressed either as GcfDNA percentage (GcfDNA/total cfDNA) or via absolute quantification in copies per milliliter (Di et al., 2021).

The gradual application of GcfDNA to clinical practice has been facilitated by the advancement of molecular detection technology (Oellerich et al., 2021). While most studies have uncovered associations between GcfDNA levels and the presence of acute rejection, a minority have failed to establish such correlations (Bromberg et al., 2017; Mayer et al., 2022). Moreover, the optimal threshold for distinguishing between rejection and non-rejection varies among studies. Although 1.0% serves as the threshold in the majority of GcfDNA investigations, some studies utilize the optimal cut-off threshold for this differentiation. Another study suggests that a lower threshold such as 0.5% may be more appropriate if a threshold is to be used (Stites et al., 2020). Furthermore, various factors, including disparities in testing methods, types of sampling tubes, and ethnicity, exert an influence on the outcomes of GcfDNA testing. Consequently, further exploration is warranted to enhance post-transplantation monitoring, curtail premature graft loss, and improve patient survival. The objective of this meta-analysis is to assess the diagnostic performance of GcfDNA as a biomarker for kidney transplant rejection. We also compared the diagnostic performance under different thresholds (1.0%/optimal cut-off threshold) and different detection methods.

## Materials and methods

### Search strategy

We conducted a comprehensive search of the PubMed, EMBASE, and Cochrane Library databases from their inception up to 30 September 2022. Retrieval was based on a combination of subject words and free words. The search terms encompassed “Donor-derived cell-free DNA,” “dd-cfDNA,” “Graft-derived cell-free DNA,” “GcfDNA,” “Kidney,” “Renal,” and “Transplantation.” These terms were utilized as either keywords or Medical Subject Headings (MeSH) terms, and different combinations of these terms were searched using Boolean operators “AND” and “OR.” Subsequently, two independent investigators (HLW and YL) conducted a secondary search of eligible studies. In cases of disagreement, consensus was reached through negotiation. The detailed search strategy is available in the Supplementary Material S1.

### Selection criteria

Following the predefined inclusion and exclusion criteria, investigators (HLW and YL) evaluated potentially relevant articles, with any discrepancies resolved by another reviewer (LW). The inclusion criteria were as follows: 1) Published studies on the use of GcfDNA for adjunctive diagnosis of rejection in kidney transplant recipients; 2) The study subjects were first-time kidney transplant recipients; 3) All episodes of rejection were confirmed by biopsy and scored using the Banff criteria; 4) The studies reported outcome indicators in the form of four-grid diagnostic tables, which could be extracted directly or indirectly. The exclusion criteria comprised: 1) Duplicate publications; 2) Reviews, conference abstracts, editorials, and case reports; 3) Animal experiments; 4) Studies involving recipients of multiorgan transplants or retransplants; 5) Studies with unavailable or incomplete data required for reconstructing a four-grid diagnostic table.

### Data extraction and quality assessment

Two independent investigators (HLW and YL) extracted relevant information, including general and clinical details from the literature (first author, publication year, country, type of rejection, study duration, study design, GcfDNA detection technology, and patient age), as well as diagnostic parameters from the literature (area under the curve [AUC], sensitivity, specificity, true positive [TP], false positive [FP], false negative [FN], true negative [TN], positive predictive value [PPV], and negative predictive value [NPV]). Quality assessment was conducted using the QUADAS-2 scale by two investigators (HLW and YL), with discrepancies resolved by a third investigator (LW) (Whiting et al., 2011).

### Statistical analysis

Data on TP, FP, TN, and FN were extracted from the included studies to establish the four values for a diagnostic 2 × 2 contingency table. We used Spearman rank correlation, Cochran’s Q test, Higgins’ I^2^ test, and forest plots to evaluate both the threshold and non-threshold sources of heterogeneity. In general, we graded the degree of heterogeneity as low (I^2^ < 25%), moderate (25% < I^2^ < 75%), or high (I^2^ > 75%) (Whiting et al., 2011). A random-effects model was employed if evidence of non-threshold effect was present. Subgroup analyses and Meta-regression were used to explore the sources of heterogeneity. Publication bias was assessed using Deeks funnel plot asymmetry test. A significance level of *p* < 0.05 was considered statistically significant. Statistical analyses were performed using Stata 15.1, Review Manager 5.4, and Meta-DiSc 1.4 (Higgins et al., 2003; Zamora et al., 2006).

## Results

### Literature search

**FIGURE 1:**
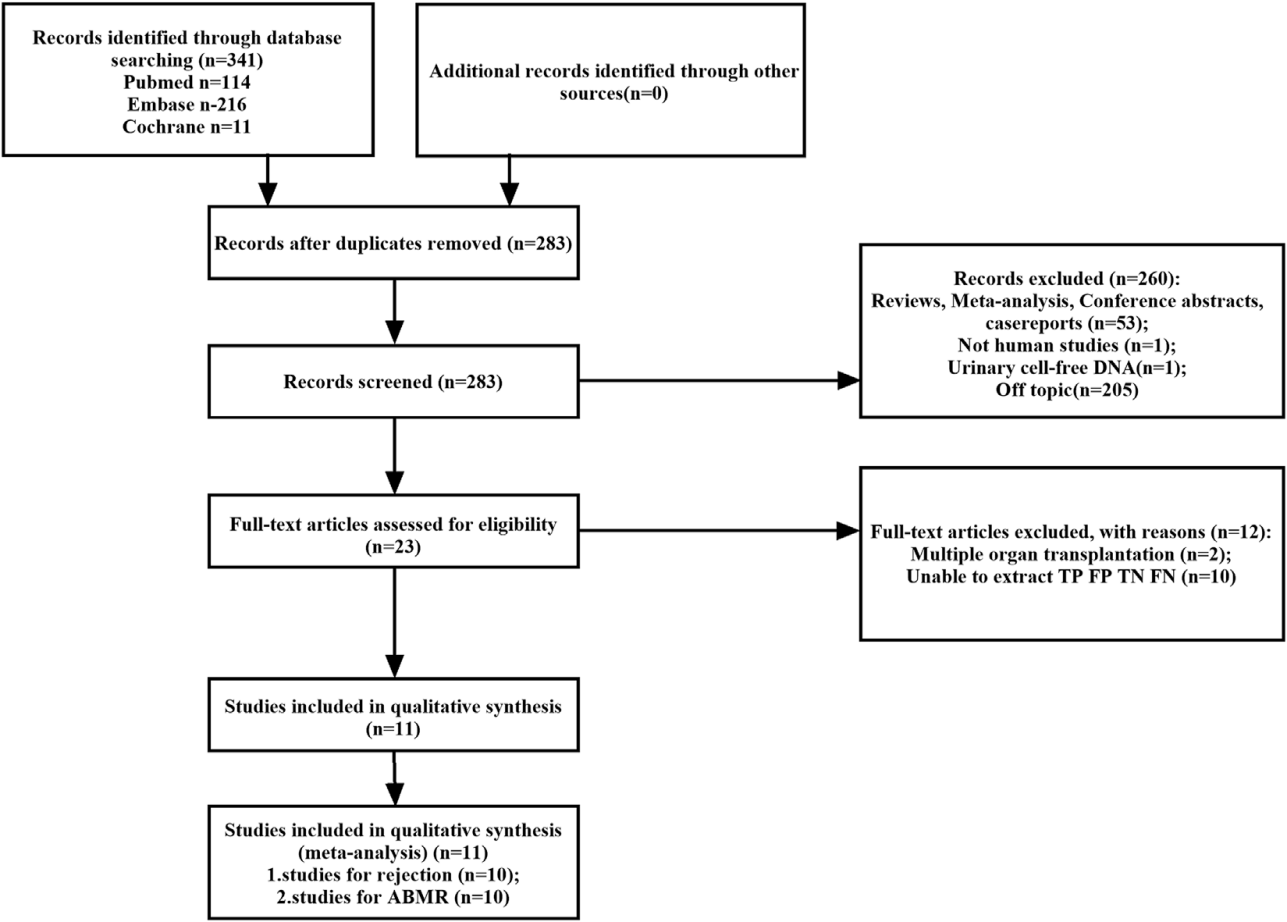
Flowchart detailing the study selection process. 11 studies that met the inclusion criteria were finally included. FN, false negative; FP, false positive; TN, true negative; TP, true positive.

A total of 341 potentially relevant studies were initially identified across the three databases. Following a comprehensive review of the full-text literature, we ultimately included 11 studies, comprising 1,248 patients, for systematic review and meta-analysis (Figure 1) (Bloom et al., 2017; Jordan et al., 2018; Sigdel et al., 2018; Huang et al., 2019; Oellerich et al., 2019; Whitlam et al., 2019; Gielis et al., 2020; Zhang et al., 2020; Puliyanda et al., 2021; Bu et al., 2022; Verhoeven et al., 2022). Detailed information pertaining to these 11 studies can be found in Supplementary Table S1. Our research encompassed studies conducted on four continents and in six different countries, including the United States (*n* = 5), Australia (*n* = 1), Belgium (*n* = 1), China (*n* = 1), Germany (*n* = 1), and the Netherlands (*n* = 1). GcfDNA content was assessed using either Next-Generation Sequencing (NGS) technology (*n* = 7) or Droplet Digital Polymerase Chain Reaction (dd-PCR) technology (*n* = 3). The study populations ranged in size from 37 to 203 patients, with an average age spanning 10–60 years. Diagnostic parameters from the literature are presented in Supplementary Table S2. Notably, there was no unified threshold for diagnosing rejection, with approximately half of the studies employing a 1% threshold. Among the included literature, 10 studies aimed to distinguish between rejection and non-rejection reactions, while 10 studies focused on diagnosing Antibody-Mediated Rejection (ABMR). Nine studies used a 1% threshold, two used a 0.5% threshold, and the remainder applied optimal cut-off thresholds tailored to each study. Separate meta-analyses were conducted for rejection and ABMR due to variations in research focus and the pathological classification of rejection.

### Data characteristics and quality assessment

Based on the QUADAS-2 assessment, Supplementary Figure S1 illustrates the quality and applicability evaluations of the 11 included studies. All of the studies met four or more of the seven criteria, indicating an overall acceptable quality level for the included studies. Nevertheless, two studies were non-consecutively enrolled and exhibited unclear inclusion and exclusion criteria. One study established a preset threshold and assessed the results against known reference standards. Moreover, two studies did not include all patients in the analysis. Concerning the inclusion of patients in the applicability analysis, two studies were categorized as “of high concern” due to a lack of detailed demographic characteristic descriptions, and one study was classified as “of unknown concern”.

### Results of the meta-analysis

#### Rejection

##### Heterogeneity analysis

**FIGURE 2:**
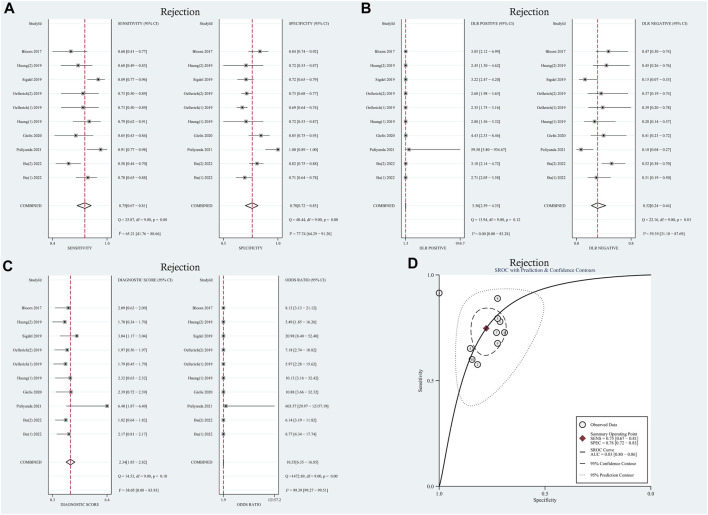
Diagnostic accuracy of GcfDNA in rejection. **(A)** Forest plots of sensitivity and specificity for GcfDNA in diagnosis. **(B)** Forest plots of the positive likelihood ratio and negative likelihood ratio in diagnosis. **(C)** Forest plots of the diagnostic odds ratio in diagnosis. **(D)** SROC curve of GcfDNA. The SROC curve analysis of the GcfDNA test accuracy in rejection diagnosis revealed an AUC of 0.83.

Moderate levels of heterogeneity were observed and assessed using the random-effects model (I^2^ > 50%) (Higgins and Green, 2011). In the entire cohorts, the results were as follows: pooled sensitivity, 0.75 (95% CI: 0.67–0.81, I^2^ = 65.21%); pooled specificity, 0.78 (95% CI: 0.72–0.83, I^2^ = 77.74%); pooled PLR, 3.36 (95% CI:2.59–4.35, I^2^ = 0.00); pooled NLR, 0.32 (95% CI: 0.24–0.44, I^2^ = 59.39); DOR, 8.77 (95% CI: 4.34–17.74, I^2^ = 99.39); AUC, 0.83 (95% CI: 0.80–0.86). Forest plots and summary receiver operating characteristics (SROC) curves are presented in Figure 2. To assess the presence of a threshold effect, the Spearman rank correlation coefficient was calculated, yielding a value of 0.24 (*p* = 0.53). Notably, the scatter plots did not exhibit the characteristic “shoulder-arms” pattern on the SROC curve, suggesting an absence of a threshold effect.

### Meta-regression analysis and subgroup analysis

To explore potential sources of heterogeneity within the included studies, we conducted both meta-regression analysis and subgroup analysis. Covariates such as study design, research center, research region, number of patients, and sampling tube were included in the meta-regression analysis. However, as summarized in Table 1, it was determined that none of these covariates could sufficiently account for the observed heterogeneity (*p* > 0.05), with the exception of the sampling tube, which demonstrated a significant contribution to the overall heterogeneity (Relative Diagnostic Odds Ratio [RDOR] = 248.68, *p* = 0.05). Given the lack of a standardized testing method and diagnostic threshold across the studies, we performed a subgroup analysis based on threshold levels and detection methods, as detailed in Table 2. The pooled sensitivity and specificity of 6 tests using the NGS detection method were 0.72 (95% CI: 0.66–0.77), 0.79 (95% CI: 0.75–0.82). Two tests used the detection method of dd-PCR, the pooled sensitivity was 0.73 (95% CI: 0.57–0.85) and the pooled specificity was 0.71 (95% CI: 0.68–0.74), respectively. Two tests used the detection method of mmPCR-NGS, the pooled sensitivity and specificity were 0.82 (95% CI: 0.71–0.90), 0.76 (95% CI: 0.70–0.82). Regarding the diagnostic threshold, 1% was employed in 5 tests, while the remaining 5 tests used optimal cut-off thresholds. The pooled sensitivity and specificity were 0.73 (95% CI: 0.67–0.79), 0.80 (95% CI: 0.76–0.83); 0.75 (95% CI: 0.68–0.82), 0.72 (95% CI: 0.69–0.75), respectively.

**TABLE 1:** Result of univariate meta-regression analysis diagnostic odd ratio.

Type	Var	*p*-value	RDOR	95% CI
Rejection	Design	0.41	0.51	0.05–4.92
	Center	0.06	0.01	0.00–1.24
	Continent	0.57	1.22	0.55–2.70
	Quality	0.88	1.08	0.23–5.15
	Tube	0.05	248.68	0.82–7,583.12
ABMR	Design	0.56	0.72	0.18–2.18
	Center	0.51	0.68	0.16–2.82
	Continent	0.22	0.53	0.17–1.69
	Quality	0.09	0.37	0.11–1.22
	Tube	0.86	1.12	0.23–5.38

Relative diagnostic odds ratio (RDOR).

**TABLE 2:** Assessment of diagnostic accuracy and heterogeneity in subgroup analysis.

Type	Parameter	Category	Number of studies	Sensitivity (95%CI)	Specificity (95%CI)	PLR(95% CI)	NLR(95% CI)	DOR (95% CI)
Rejection	All		10	0.75 (0.67–0.81)	0.78 (0.72–0.83)	3.36 (2.59–4.35)	0.32 (0.24–0.44)	8.77 (4.34–17.74)
	Method	NGS	6	0.72 (0.66–0.77)	0.79 (0.75–0.82)	2.98 (2.26–3.92)	0.37 (0.26–0.52)	8.23 (4.70–14.42)
		dd-PCR	2	0.73 (0.57–0.85)	0.71 (0.68–0.74)	2.47 (2.00–3.01)	0.40 (0.25–0.63)	6.21 (3.21–12.02)
		mmPCR-NGS	2	0.82 (0.71–0.90)	0.76 (0.70–0.82)	3.32 (2.60–4.24)	0.27 (0.10–0.72)	14.89 (7.54–29.43)
	Threshold	1%	5	0.73 (0.67–0.79)	0.80 (0.76–0.83)	3.22 (2.35–4.40)	0.32 (0.19–0.55)	10.93 (4.65–25.70)
		Optimal cut-off threshold	5	0.75 (0.68–0.82)	0.72 (0.69–0.75)	2.65 (2.26–3.09)	0.36 (0.28–0.47)	7.83 (5.21–11.76)
ABMR	All		10	0.83 (0.74–0.89)	0.75 (0.70–0.80)	3.37 (2.64–4.30)	0.23 (0.15–0.36)	14.65 (7.94–27.03)
	Method	NGS	7	0.79 (0.72–0.84)	0.74 (0.70–0.77)	3.15 (2.36–4.20)	0.33 (0.22–0.47)	10.28 (5.67–18.65)
		dd-PCR	3	0.85 (0.71–0.94)	0.75 (0.63–0.84)	3.04 (2.03–4.54)	0.25 (0.13–0.46)	12.77 (5.14–31.71)
	Threshold	1%	5	0.76 (0.67–0.83)	0.78 (0.74–0.83)	3.32 (2.57–4.29)	0.33 (0.22–0.48)	10.13 (5.76–17.81)
		Optimal cut-off threshold	5	0.85 (0.76–0.91)	0.68 (0.62–0.73)	2.89 (2.01–4.14)	0.29 (0.18–0.48)	11.19 (4.62–27.09)

Next-generation sequencing (NGS); digital droplet polymerase chain reaction (ddPCR); antibody-mediated rejection (ABMR).

### Sensitivity analysis

We conducted a sensitivity analysis to assess the impact of each individual study on the overall outcomes of the meta-analysis. The results of this analysis, presented in Supplementary Figure S2A, suggest that the stability and reliability of the included literature were acceptable.

### Differential diagnostic value of GcfDNA

A random-effects model was used for the meta-analysis of included studies. Results showed a pooled sensitivity of 0.75 (95% CI: 0.67–0.81), pooled specificity of 0.78 (95% CI: 0.72–0.83), pooled positive likelihood ratio (PLR) of 3.36 (95% CI: 2.59–4.35), pooled negative likelihood ratio (NLR) of 0.32 (95% CI: 0.24–0.44), diagnostic odds ratio (DOR) of 8.77 (95% CI: 4.34–17.74). Hierarchical summary receiver operating characteristic (HSROC) curves are shown in Supplementary Figure S3A. The estimated value of β was −0.25 (95% CI: 1.39–0.89), and the value of z and the *p*-value were −0.43 and 0.67 separately, indicates that the SROC curve was symmetric. The diagnostic accuracy of GcfDNA was 0.83, *p* < 0.05. Based on the aforementioned data, it can be concluded that GcfDNA exhibits favorable sensitivity and specificity for the early diagnosis of rejection, substantiating its diagnostic value. These results collectively suggest that GcfDNA demonstrates acceptable diagnostic performance for the detection of rejection. Furthermore, an assessment of publication bias in the selected studies was conducted using the Deeks’ funnel plot asymmetry test (Supplementary Figure S4A), which did not indicate any significant publication bias (*p* = 0.59).

### ABMR

#### Heterogeneity analysis

**FIGURE 3:**
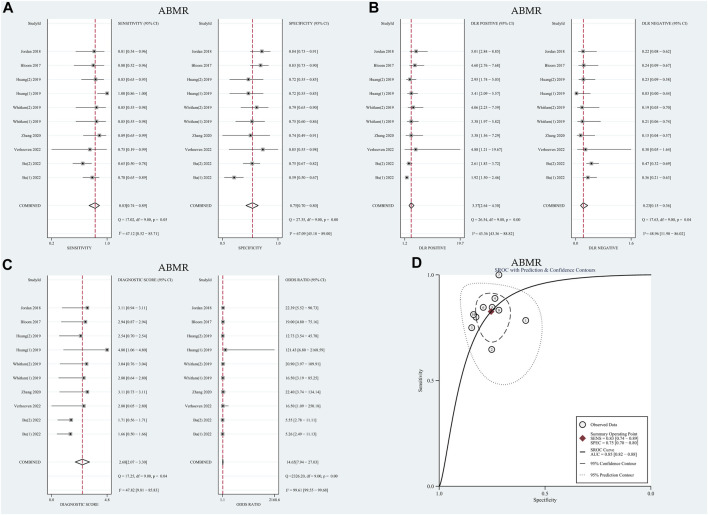
Diagnostic accuracy of GcfDNA in ABMR. **(A)** Forest plots of sensitivity and specificity for GcfDNA in diagnosis. **(B)** Forest plots of the positive likelihood ratio and negative likelihood ratio in diagnosis. **(C)** Forest plots of the diagnostic odds ratio in diagnosis. **(D)** SROC curve of GcfDNA. The SROC curve analysis of the GcfDNA test accuracy in rejection diagnosis revealed an AUC of 0.84.

The I^2^ values for sensitivity and specificity were found to be 47.12% and 67.09% (*p* < 0.01), respectively. These values suggest moderate levels of heterogeneity, leading to the calculation of sensitivity and specificity using the random effects model. The pooled sensitivity and specificity were determined to be 0.83 (95% CI: 0.74–0.89) and 0.75 (95% CI: 0.70–0.80), respectively. Furthermore, the pooled positive likelihood ratio (PLR), negative likelihood ratio (NLR), and diagnostic odds ratio (DOR) were calculated as 3.37 (95% CI: 2.64–4.30), 0.23 (95% CI: 0.15–0.36), and 14.65 (95% CI: 7.94–27.03), respectively. The AUC in the SROC curve was determined to be 0.85 (95% CI: 0.82–0.88), as illustrated in Figure 3. Similarly, there was no threshold effect: the spearman correlation coefficient was 0.319 (*p* = 0.40) and the scatter plots did not appear as “shoulder-arms” in the image formed on the SROC curve.

### Meta-regression analysis and subgroup analysis

Similarly, meta-regression and subgroup analysis were conducted to further explore potential sources of heterogeneity. As summarized in Table 1, it was observed that none of the covariates could account for the observed heterogeneity (*p* > 0.05). Subsequently, subgroup analysis was carried out based on threshold levels and detection methods, as detailed in Table 2. For the 7 tests utilizing the NGS detection method, the pooled sensitivity and specificity were determined to be 0.79 (95% CI: 0.72–0.84) and 0.74 (95% CI: 0.70–0.77), respectively. On the other hand, among the 3 tests employing the dd-PCR detection method, the pooled sensitivity was 0.85 (95% CI: 0.71–0.94), and the pooled specificity was 0.75 (95% CI: 0.63–0.84).

### Sensitivity analysis

The results of the sensitivity analysis demonstrated the overall stability of the included literature, as illustrated in Supplementary Figure S2B.

### Differential diagnostic value of GcfDNA

The meta-analysis, conducted using a random-effects model, revealed that the combined pooled sensitivity, specificity, PLR, NLR, DOR, and the area under the SROC curve of the 10 tests were 0.83 (95% CI: 0.74–0.89), 0.75 (95% CI: 0.70–0.80), 3.37 (95% CI: 2.64–4.30), 0.23 (95% CI: 0.15–0.36), 14.65 (95% CI: 7.94–27.03), and 0.85 (95% CI: 0.82–0.88), respectively, as depicted in Figure 3. The HSROC curves are presented in Supplementary Figure S3B. The estimated value of β was −0.39 (95% CI: 1.61–0.84), with the corresponding values of z and P being −0.62 and 0.54, respectively, indicating a symmetric SROC curve. The diagnostic accuracy of GcfDNA was 0.85, with *p* < 0.05. Therefore, it can be concluded that GcfDNA exhibits excellent discriminatory value for ABMR. The assessment of publication bias using the Deeks funnel plot indicated no significant publication bias (*p* = 0.07), as shown in Supplementary Figure S4B.

## Discussion

GcfDNA, as an emerging biomarker, holds significant clinical relevance for the early prediction of rejection (Lo et al., 1998). Nevertheless, several critical issues warrant discussion, primarily pertaining to diagnostic thresholds and detection methods. In this meta-analysis, we offer comprehensive insights into the distinctive diagnostic value of GcfDNA concerning kidney allograft rejection and ABMR.

This comprehensive meta-analysis encompassed 20 tests conducted between 2017 and 2022, collectively involving 1,248 patients. Our findings reveal that GcfDNA exhibits comparable diagnostic accuracy for both rejection and ABMR scenarios. Specifically, the pooled sensitivity, specificity, and the area under the SROC curve were 0.75 (95% CI: 0.67–0.81), 0.78 (95% CI: 0.72–0.83), and 0.83 (95% CI: 0.80–0.86) for rejection, and 0.83 (95% CI: 0.74–0.89), 0.75 (95% CI: 0.70–0.80), and 0.85 (95% CI: 0.82–0.88) for ABMR, respectively. The diagnostic sensitivity of ABMR alone is higher, which confirms the research of Bloom et al. (2017). Taking a step furtherGcfDNA elevation has often been demonstrated in patients with ABMR, its association with TCMR is less clear (Agbor-Enoh et al., 2018; Agbor-Enoh, 2019; Wijtvliet et al., 2020). One possible explanation is that microvascular injury is the main pathological change of ABMR, while TCMR is characterized by interstitial inflammation and tubulitis, only the higher-level classification releases GcfDNA (Haas et al., 2018). Therefore only a small increase in GcfDNA levels was observed in patients with TCMR (Bloom et al., 2017). This may account for the limited sensitivity of GcfDNA in identifying TCMR. However, the conclusion remains inconclusive. In recipients with acute rejection or without rejection, the GcfDNA (%) detected by short amplicons (86–128 bp) was significantly higher than that quantified with long amplicons (106–156 bp). In the study by Huang et al., the median GcfDNA (%) in patients with TCMR measured using the Allosure detection method was even lower than that of patients without rejection (0.27% vs. 0.38%). The amplicon length was 100–130 bp (Huang et al., 2019). Sigdel et al. successfully used the multiplex PCR NGS methodology to detect plasma dd-cfDNA elevations in patients with TCMR (Sigdel et al., 2018). A group also observed an increase in GcfDNA levels of patients with TCMR through ddPCR detection. In order to correct each sample for its individual mean fragment length a ddPCR assay using amplicons of two different lengths (94 bp and 249 bp) was developed (Oellerich et al., 2019). This seems to implicate that the research results are influenced by the quantification methodology used (Dauber et al., 2020). Further research is required to test this hypothesis. As mentioned above, there are still many questions about the identification of TCMR using GcfDNA, and more research on TCMR related analysis is needed for further analysis.

It is important to note that a moderate level of heterogeneity was observed in sensitivity and specificity across the included studies. To identify potential sources of this heterogeneity, we conducted meta-regression analyses, taking into account various factors such as study design, research center, research region, number of patients, and the type of collection tube utilized. Unfortunately, the meta-regression did not provide clear insights into the origins of this heterogeneity. Notably, among the covariates studied, the type of collection tube appeared to contribute the most to the observed heterogeneity. In detail, the tube yielded maximal RDOR value and minimal *p*-value among all covariates. It is crucial to emphasize that the choice of collection tubes employed by different research centers has not been standardized. Maintaining sample stability is paramount to ensure accurate cfDNA analysis. Furthermore, the rupture of white blood cells (WBCs) within the collection tube can lead to the release of cfDNA, resulting in an elevated DNA background that may impact cfDNA detection results. Streck tubes, designed to stabilize blood cells and prevent hemolysis and WBC degradation over time, offer a solution to this issue. Research by Nikolaev et al. demonstrated that cfDNA in blood samples stored in EDTA tubes becomes contaminated with DNA fragments from lysed leukocytes after 16 h at room temperature, whereas samples stored in Streck tubes remain uncontaminated for at least 7 days (Nikolaev et al., 2018). This underscores the importance of using Streck tubes to prevent leukocyte lysis and maintain the stability of blood samples (Lam et al., 2004; Medina Diaz et al., 2016; Di et al., 2021).

We subsequently conducted a subgroup analysis, stratifying the data based on threshold levels and detection methods, in an effort to discern the origin of the observed heterogeneity. Our findings reveal intriguing insights into the impact of different thresholds and detection methods on diagnostic performance. For the diagnosis of rejection, the specificity associated with the 1% threshold was notably higher (0.80) compared to the optimal cut-off threshold (0.72). Moreover, the corresponding DOR value was also higher at 10.93. It is worth highlighting that approximately half of the studies included in our analysis employed the 1% threshold as the criterion for diagnosing rejection, while the remaining studies opted for the optimal cut-off threshold. The question of whether the 1% threshold can be universally adopted as the standard for diagnosing rejection remains open for further verification. Conversely, in the context of diagnosing ABMR, the optimal cut-off threshold exhibited superior sensitivity (0.85 vs. 0.75) and a higher DOR value (11.19 vs. 8.88) when compared to the 1% threshold. These findings align with a study conducted by Huang et al., which reported higher sensitivity (100% vs. 83.3%), specificity (71.8% vs. 71.8%), PPV (68.6% vs. 64.5%), and NPV (100% vs. 87.5%) for the optimal cut-off threshold when compared to the 1% threshold (Huang et al., 2019). These results underscore the importance of carefully considering the threshold level when utilizing GcfDNA for diagnosing rejection and ABMR in kidney transplant recipients, as it can significantly impact the diagnostic accuracy and clinical utility of this biomarker. Further research and standardization efforts are warranted to establish the most appropriate thresholds for different clinical scenarios.

Two distinct methods are employed for establishing the cut-off threshold of GcfDNA: percentage or copy quantity. The GcfDNA percentage signifies the relative proportion of graft cfDNA in plasma, calculated as the ratio of graft cfDNA to total cfDNA. It is important to note that the majority of cfDNA originates from circulating WBCs, and the GcfDNA percentage may be influenced by fluctuations in both graft and recipient cfDNA levels. These fluctuations can arise due to various factors such as graft quality, leukopenia, leukocytosis, among others (Sun et al., 2015; Schütz et al., 2017). A notable study conducted by Oellerich et al. yielded intriguing insights into the quantification methods for GcfDNA thresholds. They observed that the diagnostic accuracy, as measured by the Area Under the Curve (AUC), of absolute quantification in distinguishing acute rejection confirmed by biopsy (AUC = 0.83) was significantly superior to that of GcfDNA percentage (AUC = 0.73), with a statistically significant difference (*p* < 0.01) (Oellerich et al., 2019). These findings suggest that absolute quantification of GcfDNA holds promising potential as a more accurate and reliable method for diagnostic purposes. The choice between GcfDNA percentage and absolute quantification for establishing thresholds warrants careful consideration, as it can significantly impact the diagnostic precision and clinical utility of GcfDNA in assessing kidney transplant rejection. Further research and consensus-building efforts are essential to determine the most appropriate quantification method for different clinical scenarios.

Notably, the detection method mmPCR-NGS exhibited a higher pooled sensitivity compared to NGS and ddPCR, boasting a sensitivity rate of 0.82. Furthermore, the Diagnostic Odds Ratio (DOR) associated with mmPCR-NGS was the most favorable among the three methods, with a DOR of 14.89. This suggests that the utilization of Multiplex PCR for detecting Single Nucleotide Polymorphisms (SNPs) in conjunction with NGS can yield more precise and sensitive results. This combination has previously been employed successfully by Enyedi et al. in the more accurate detection of BRCA1 and BRCA2 genes (Enyedi et al., 2016). When diagnosing ABMR, ddPCR demonstrated superior sensitivity, specificity, and DOR values. But due to a smaller number of studies and patients, more studies and further laboratory studies may be needed to confirm this phenomenon. It is worth mentioning that similar performance metrics have been reported for ddPCR and NGS methods (Oellerich et al., 2020). Moreover, ddPCR offers a much broader linear quantifiable range, spanning from 0.15% to 99.9%, in contrast to the more limited range of targeted NGS, which typically spans from 0.20% to 20%. (Basu, 2017; Oellerich et al., 2020). In terms of application, if GcfDNA is used as an indicator for long-term monitoring of grafts, NGS is clearly expensive (Watkins and Charames, 2018) and time-consuming (2-3days vs. 1d), although there are 3 commercially available tests (AlloSure Kidney, TRAC Kidney, and Prospera). However, so far, the validation of these methods has been limited, and caution should be exercised when using liquid biopsy results to guide clinical practices (Whitlam et al., 2019).

There are limitations within this meta-analysis, and the results should be interpreted with caution. First, in order to improve the quality of the literature, some data such as conference abstracts, case studies, and other unpublished literature were excluded. All of these inevitably increase publication bias to a certain extent. Second, the overall sample size included in the study was small, limiting the interpretation of the results. Heterogeneity was observed in the pooled sensitivity and specificity of rejection and ABMR analysis, although meta-regression analysis and subgroup analysis were conducted to explore potential sources of heterogeneity before conducting the meta-analysis.

## Conclusion

In summary, the collective findings underscore the substantial diagnostic potential of GcfDNA as a biomarker for discriminating between rejection and Antibody-Mediated Rejection (ABMR) in kidney transplant recipients. Notably, the optimal cut-off threshold exhibits a particularly favorable diagnostic performance in the context of ABMR diagnosis. Furthermore, it is worth highlighting that the accuracy of the ddPCR detection method in diagnosing ABMR surpasses that of Next-Generation Sequencing (NGS). However, it is imperative to acknowledge that further extensive and comprehensive studies are warranted to corroborate and build upon these observations.

These results hold significant promise and implications for the field of kidney transplantation, potentially offering improved diagnostic capabilities and guiding more tailored therapeutic interventions. Nevertheless, the ongoing pursuit of robust and validated methodologies remains paramount to harness the full potential of GcfDNA as a diagnostic tool in this context.

## Data Availability

The original contributions presented in the study are included in the article/Supplementary Material, further inquiries can be directed to the corresponding author.
