# Supplementary material for: Diagnostic performance of GcfDNA in kidney allograft rejection: a meta-analysis
Source: Front Physiol. 2024 Jan 9;14:1293402. doi: 10.3389/fphys.2023.1293402 (PMC10803602; doi:10.3389/fphys.2023.1293402)
Supplement: Supplementary file 1 [file Table1.DOCX]

Table 1 Result of univariate meta-regression analysis diagnostic odd ratio

| **Type** | **Var** | **P-value** | **RDOR** | **95% CI** |
| --- | --- | --- | --- | --- |
| Rejection | Design | 0.41 | 0.51 | 0.05-4.92 |
|  | Center | 0.06 | 0.01 | 0.00-1.24 |
|  | Continent | 0.57 | 1.22 | 0.55-2.70 |
|  | Quality | 0.88 | 1.08 | 0.23-5.15 |
|  | Tube | 0.05 | 248.68 | 0.82-7583.12 |
| ABMR | Design | 0.56 | 0.72 | 0.18-2.18 |
|  | Center | 0.51 | 0.68 | 0.16-2.82 |
|  | Continent | 0.22 | 0.53 | 0.17-1.69 |
|  | Quality | 0.09 | 0.37 | 0.11-1.22 |
|  | Tube | 0.86 | 1.12 | 0.23-5.38 |

relative diagnostic odds ratio (RDOR)
