# Supplementary material for: Diagnostic performance of GcfDNA in kidney allograft rejection: a meta-analysis
Source: Front Physiol. 2024 Jan 9;14:1293402. doi: 10.3389/fphys.2023.1293402 (PMC10803602; doi:10.3389/fphys.2023.1293402)
Supplement: Supplementary file 2 [file Table2.DOCX]

Table 2 Assessment of diagnostic accuracy and heterogeneity in subgroup analysis

| **Type** | **Parameter** | **Category** | **Number of studies** | Sensitivity（95%CI） | Specificity（95%CI） | **PLR(95% CI)** | **NLR(95% CI)** | **DOR(95% CI)** |
| --- | --- | --- | --- | --- | --- | --- | --- | --- |
| Rejection | All |  | 10 | 0.75 (0.67-0.81) | 0.78 (0.72-0.83) | 3.36 (2.59-4.35) | 0.32 (0.24-0.44) | 8.77 (4.34-17.74) |
|  | Method | NGS | 6 | 0.72 (0.66-0.77) | 0.79 (0.75-0.82) | 2.98 (2.26-3.92) | 0.37 (0.26-0.52) | 8.23(4.70-14.42) |
|  |  | dd-PCR | 2 | 0.73 (0.57-0.85) | 0.71 (0.68-0.74) | 2.47 (2.00-3.01) | 0.40 (0.25-0.63) | 6.21 (3.21-12.02) |
|  |  | mmPCR-NGS | 2 | 0.82 (0.71-0.90) | 0.76 (0.70-0.82) | 3.32 (2.60-4.24) | 0.27 (0.10-0.72) | 14.89 (7.54-29.43) |
|  | Threshold | 1% | 5 | 0.73 (0.67-0.79) | 0.80 (0.76-0.83) | 3.22 (2.35-4.40) | 0.32 (0.19-0.55) | 10.93 (4.65-25.70) |
|  |  | Optimal cut-off threshold | 5 | 0.75 (0.68-0.82) | 0.72 (0.69-0.75) | 2.65 (2.26-3.09) | 0.36 (0.28-0.47) | 7.83 (5.21-11.76) |
| ABMR | All |  | 10 | 0.83 (0.74-0.89) | 0.75 (0.70-0.80) | 3.37 (2.64-4.30) | 0.23 (0.15-0.36) | 14.65 (7.94-27.03) |
|  | Method | NGS | 7 | 0.79 (0.72-0.84) | 0.74 (0.70-0.77) | 3.15 (2.36-4.20) | 0.33 (0.22-0.47) | 10.28 (5.67-18.65) |
|  |  | dd-PCR | 3 | 0.85 (0.71-0.94) | 0.75 (0.63-0.84) | 3.04 (2.03-4.54) | 0.25 (0.13-0.46) | 12.77 (5.14-31.71) |
|  | Threshold | 1% | 5 | 0.76 (0.67-0.83) | 0.78 (0.74-0.83) | 3.32 (2.57-4.29) | 0.33(0.22-0.48) | 10.13 (5.76-17.81) |
|  |  | Optimal cut-off threshold | 5 | 0.85 (0.76-0.91) | 0.68 (0.62-0.73) | 2.89 (2.01-4.14) | 0.29 (0.18-0.48) | 11.19 (4.62-27.09) |

next-generation sequencing (NGS); digital droplet polymerase chain reaction (ddPCR); antibody-mediated rejection (ABMR).
