# Supplementary material for: Diagnostic performance of GcfDNA in kidney allograft rejection: a meta-analysis
Source: Front Physiol. 2024 Jan 9;14:1293402. doi: 10.3389/fphys.2023.1293402 (PMC10803602; doi:10.3389/fphys.2023.1293402)
Supplement: Supplementary file 3 [file DataSheet2.DOCX]

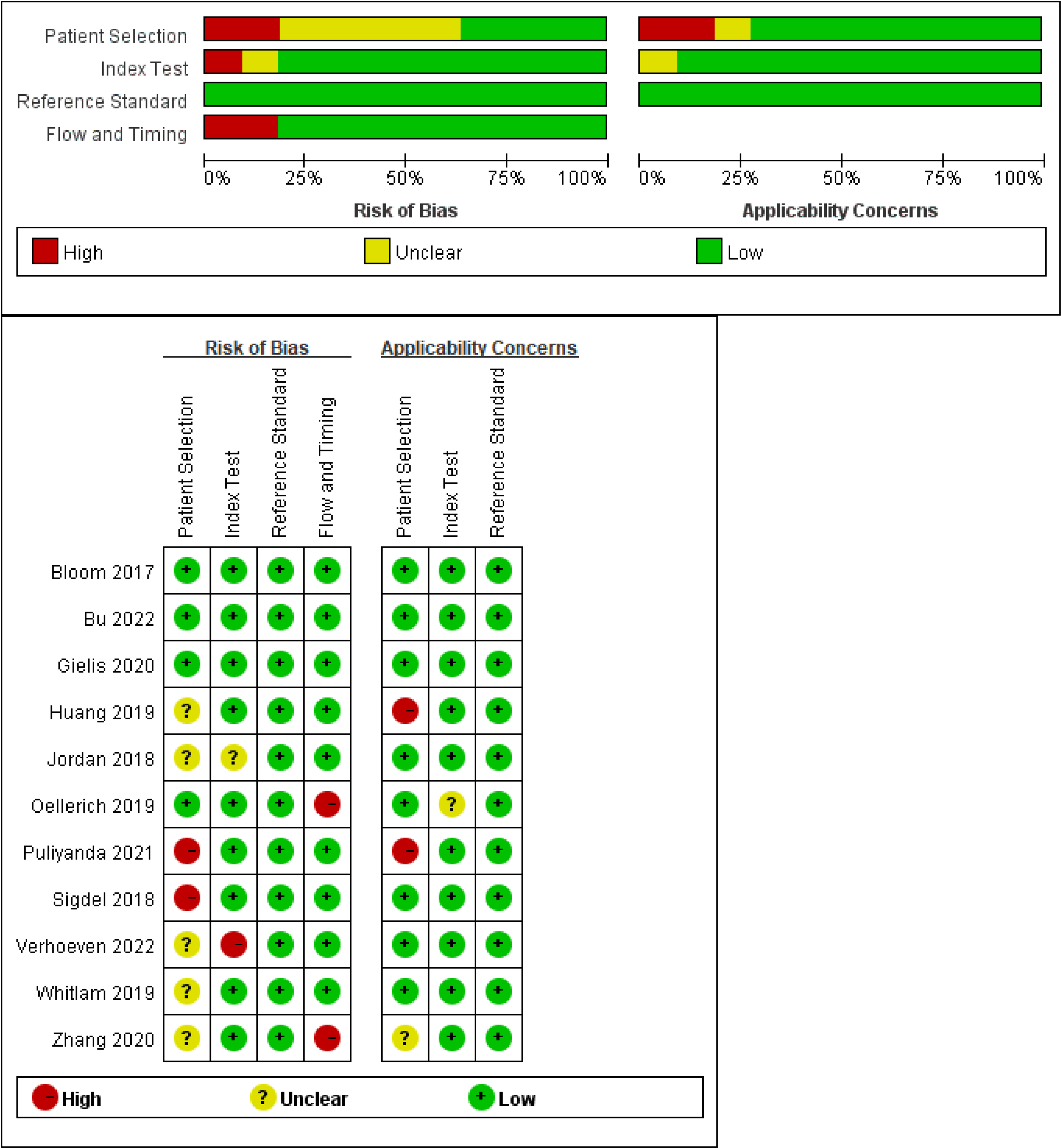


Figure S1 Quality evaluation plot of methodological.


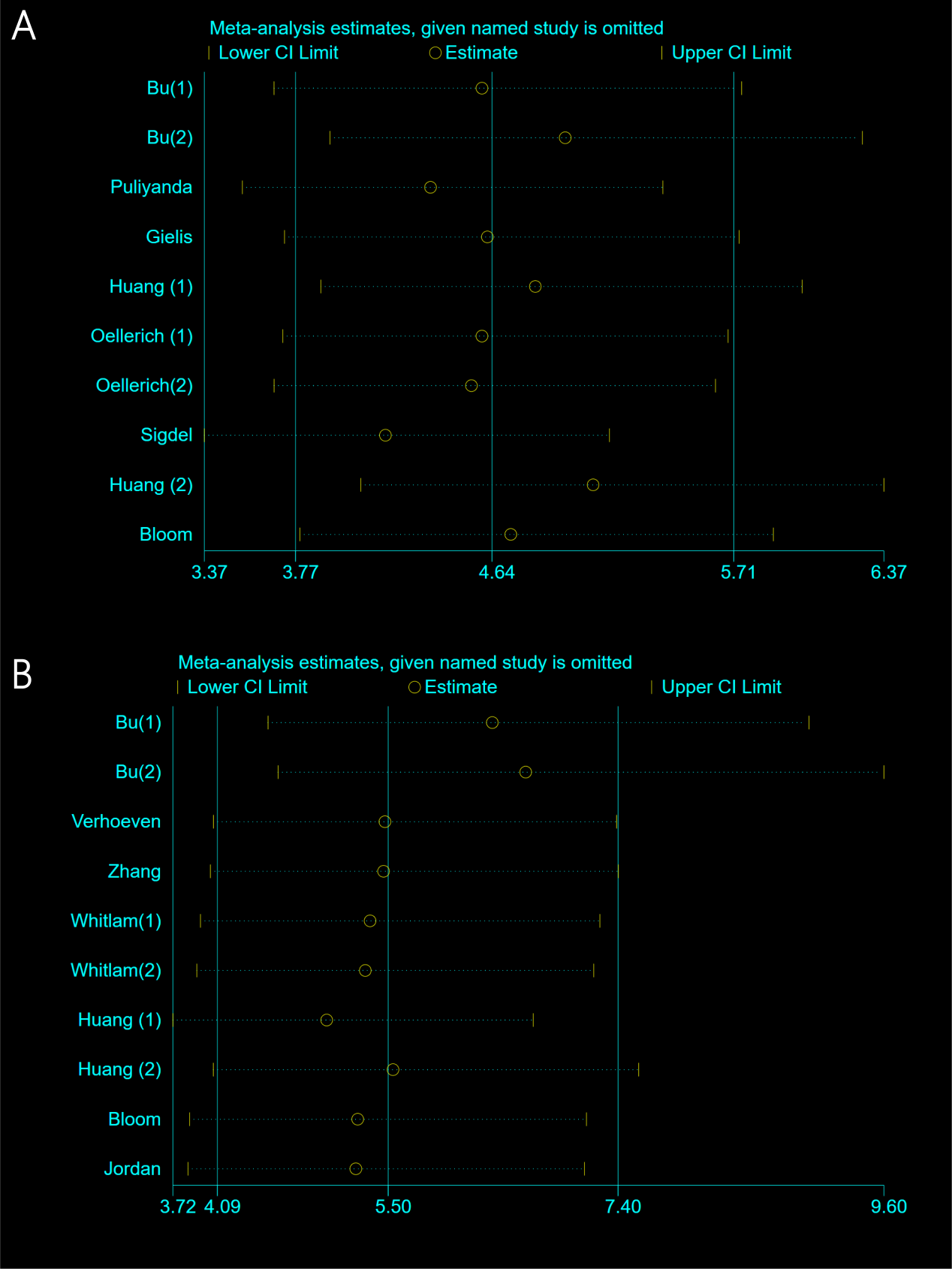


Figure S2 The influence of each study on the outcome of the meta-analysis. (A) Sensitivity analysis of rejection; (B) Sensitivity analysis of ABMR.


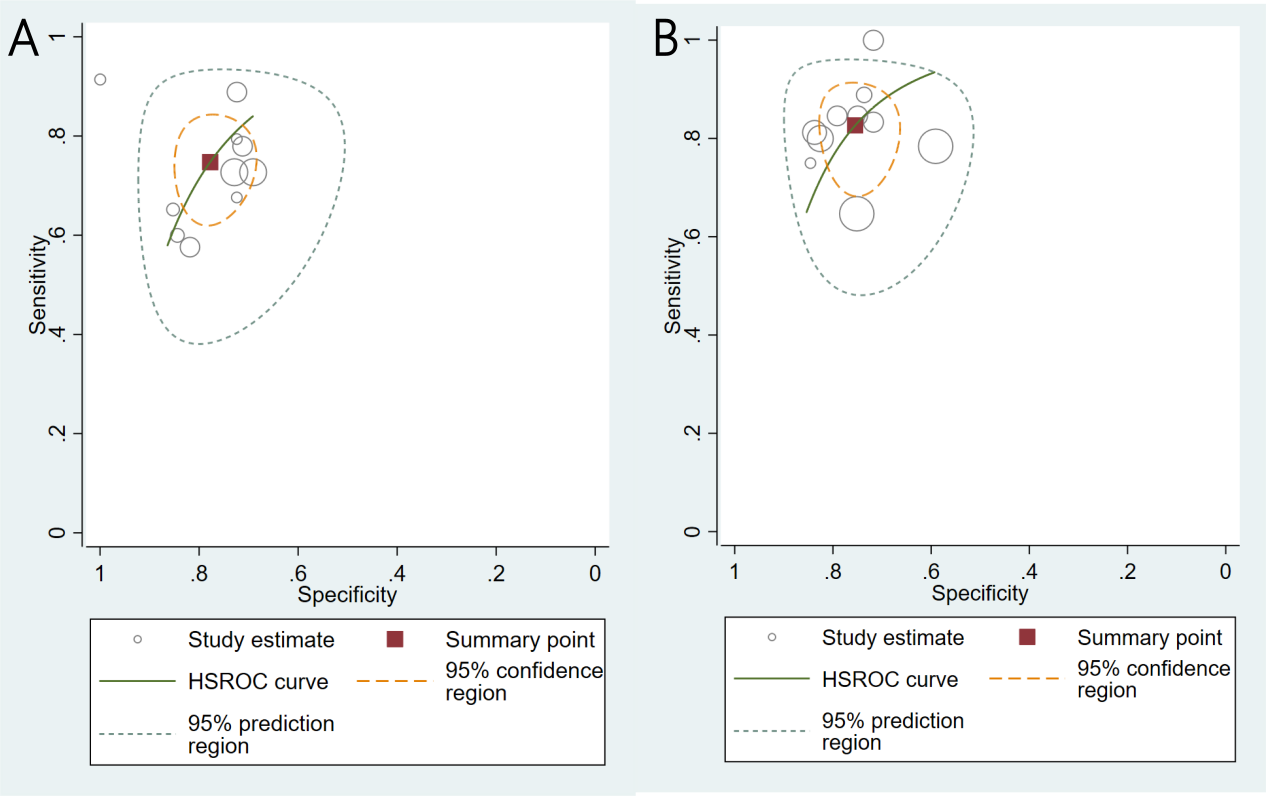


Figure S3 The HSROC curve of the differential diagnostic value of rejection (A) and ABMR (B).


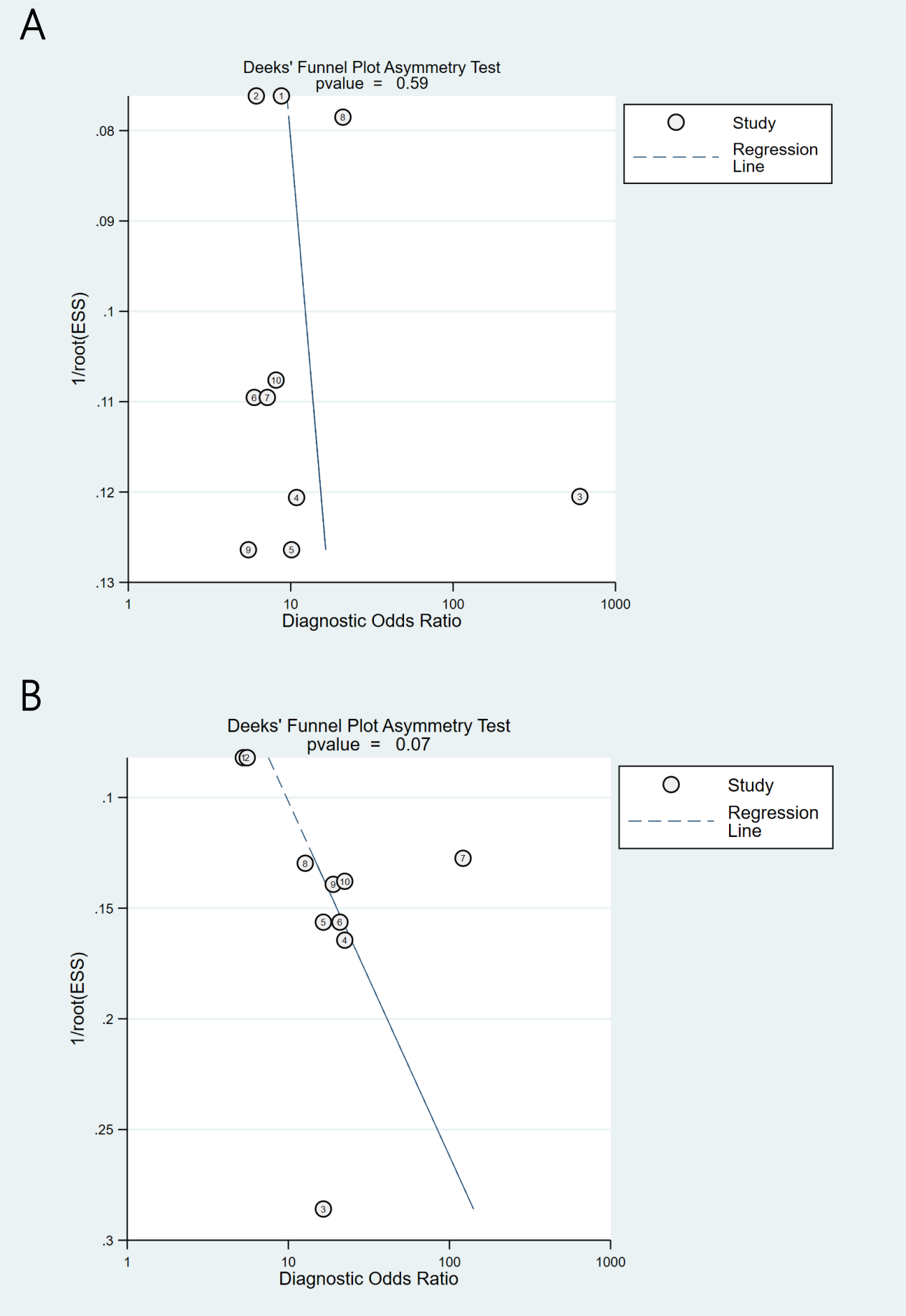


Figure S4 Deeks’ funnel plot asymmetry test of GcfDNA for diagnosis of rejection (A) and ABMR (B).
