# Supplementary material for: Diagnostic performance of GcfDNA in kidney allograft rejection: a meta-analysis
Source: Front Physiol. 2024 Jan 9;14:1293402. doi: 10.3389/fphys.2023.1293402 (PMC10803602; doi:10.3389/fphys.2023.1293402)
Supplement: Supplementary file 4 [file DataSheet1.DOCX]

**The detailed search strategy**

PubMed (MedLine):

#1 Search: ((((((((((((((dd-cfDNA) OR (dd-cf DNA)) OR (Gcf-DNA)) OR (GcfDNA)) OR (donor-derive cell-free DNA)) OR (Graft-derived cell-free DNA))) OR (ddcfDNA)) OR (donor-derived cell-free DNA)) OR (donor derive cell free DNA)) OR (donor derived cell free DNA)) OR (Graft derived cell free DNA)) OR (Graft derive cell-free DNA)) OR (Graft derive cell free DNA)) Sort by: Publication Date 927

#2 Search: "Kidney Transplantation"[Mesh] Sort by: Publication Date 104,880

#3 ((((((((Renal Transplantation[Title/Abstract]) OR (Renal Transplantations[Title/Abstract])) OR (Transplantations, Renal[Title/Abstract])) OR (Transplantation, Renal[Title/Abstract])) OR (Grafting, Kidney[Title/Abstract])) OR (Kidney Grafting[Title/Abstract])) OR (Transplantation, Kidney[Title/Abstract])) OR (Kidney Transplantations[Title/Abstract])) OR (Transplantations, Kidney[Title/Abstract]) Sort by: Publication Date 29,939

#4 ("Kidney Transplantation"[Mesh]) OR (((((((((Renal Transplantation[Title/Abstract]) OR (Renal Transplantations[Title/Abstract])) OR (Transplantations, Renal[Title/Abstract])) OR (Transplantation, Renal[Title/Abstract])) OR (Grafting, Kidney[Title/Abstract])) OR (Kidney Grafting[Title/Abstract])) OR (Transplantation, Kidney[Title/Abstract])) OR (Kidney Transplantations[Title/Abstract])) OR (Transplantations, Kidney[Title/Abstract])) Sort by: Publication Date 110,478

#5 Search: (("Kidney Transplantation"[Mesh]) OR (((((((((Renal Transplantation[Title/Abstract]) OR (Renal Transplantations[Title/Abstract])) OR (Transplantations, Renal[Title/Abstract])) OR (Transplantation, Renal[Title/Abstract])) OR (Grafting, Kidney[Title/Abstract])) OR (Kidney Grafting[Title/Abstract])) OR (Transplantation, Kidney[Title/Abstract])) OR (Kidney Transplantations[Title/Abstract])) OR (Transplantations, Kidney[Title/Abstract]))) AND (((((((((((((((dd-cfDNA) OR (dd-cf DNA)) OR (Gcf-DNA)) OR (GcfDNA)) OR (donor-derive cell-free DNA)) OR (Graft-derived cell-free DNA))) OR (ddcfDNA)) OR (donor-derived cell-free DNA)) OR (donor derive cell free DNA)) OR (donor derived cell free DNA)) OR (Graft derived cell free DNA)) OR (Graft derive cell-free DNA)) OR (Graft derive cell free DNA))) Sort by: Publication Date 114

EmBase:

#1 'kidney transplantation'/exp 177587

#2 'renal transplantation':ab,ti OR 'renal transplantations':ab,ti OR 'transplantations, renal':ab,ti OR 'transplantation, renal':ab,ti OR 'grafting, kidney':ab,ti OR 'kidney grafting':ab,ti OR 'transplantation, kidney':ab,ti OR 'kidney transplantations':ab,ti OR 'transplantations, kidney':ab,ti 42056

#3 'dd-cf dna':ab,ti OR 'dd-cfdna':ab,ti OR 'ddcfdna':ab,ti OR 'gcf-dna':ab,ti OR 'gcfdna':ab,ti OR 'gcf dna':ab,ti OR 'donor-derive cell-free dna':ab,ti OR 'donor-derive cell free dna':ab,ti OR 'donor derive cell free dna':ab,ti OR 'graft-derived cell-free dna':ab,ti OR 'graft derived cell free dna':ab,ti OR 'graft derive cell free dna':ab,ti OR 'graft derive cell-free dna':ab,ti 501

#4 #1 OR #2 180974

#5 #3 AND #4 216

Cochraen Library:

#1 MeSH descriptor: [Kidney Transplantation] explode all trees 3721

#2 ((Renal Transplantation):ti,ab,kw OR (Renal Transplantations):ti,ab,kw OR (Transplantations, Renal):ti,ab,kw OR (Transplantation, Renal):ti,ab,kw OR (Grafting, Kidney):ti,ab,kw OR (Kidney Grafting):ti,ab,kw OR (Transplantation, Kidney):ti,ab,kw OR (Kidney Transplantations):ti,ab,kw OR (Transplantations, Kidney)):ti,ab,kw (Word variations have been searched) 15359

#3 #1 OR #2 15359

#4 ((dd-cf DNA):ti,ab,kw OR (ddcfDNA):ti,ab,kw OR (dd-cfDNA):ti,ab,kw OR (donor-derive cell-free DNA):ti,ab,kw OR (donor-derived cell-free DNA):ti,ab,kw OR (GcfDNA):ti,ab,kw OR (Gcf DNA):ti,ab,kw OR (Gcf-DNA):ti,ab,kw OR (donor derive cell free DNA):ti,ab,kw OR(Graft derived cell free DNA):ti,ab,kw OR (Graft derive cell-free DNA):ti,ab,kw OR(Graft derive cell free DNA)):ti,ab,kw (Word variations have been searched) 53

#5 #3 AND #4 11

**Table S1 Characteristics of the included studies**

| **Number** | **Reference** | **Year** | **Country** | **Study period** | **Design** | **Age** | **Method** | **Number of patients** | **Number of samples** | **Reference standard** | **Specific types of rejection** |
| --- | --- | --- | --- | --- | --- | --- | --- | --- | --- | --- | --- |
| 1 | Bu | 2022 | United States | 2016.06-2020.01 | Pro-Co | 50 (17-84) | NGS | 203 | 219 | 2019 | Rejection: ABMR, TCMR, combined ABMR and TCMR ABMR：ABMR, Mixed rejection |
| 2 | Gielis | 2019 | Belgium | 2014.10-2017.03 | Pro-Co | 51 (18-71) | mmPCR-NGS | 107 | 91 | 2013 | rejection: borderline treated rejection episodes, TCMR, ABMR, combined TCMR and ABMR |
| 3 | Whitlam | 2019 | Australia | N/A | Pro-Co | 53 (39-61) | ddPCR | 55 | 61 | 2013 | ABMR: composite diagnosis of acute antibody mediated rejection and chronic active antibody mediated rejection |
| 4 | Sigdel | 2019 | United States | N/A | Ret-Co | 49 (23-76) | mmPCR-NGS | 178 | 217 | 2017 | rejection: ABMR, ABMR combined TCMR, TCMR |
| 5 | Huang | 2019 | United States | 2017.08-2018.09 | N/A | 49 (N/A) | NGS | N/A | 63 | 2013 | rejection: CMR, ABMR, or mixed rejection, ABMR, and CMR ABMR: isolated ABMR and mixed CMR/ABMR |
| 6 | Bloom | 2017 | United States | 2015.04-2016.05 | Pro-Co | 46 (N/A) | NGS | 102 | 107 | 2013 and 2017 | rejection: TCMR, acute/active ABMR, and chronic, active ABMR ABMR: acute/active ABMR, and chronic, active ABMR |
| 7 | Oellerich | 2019 | Germany | 2013.09-2017.10 | Pro-Co | 52 (N/A) | ddPCR | 189 | 417 | 2017 | rejection: Acute TCMR, AMR, Chronic Active AMR, mixed Acute TCMR, and Active AMR |
| 8 | Puliyanda | 2021 | United States | 2017.10-2019.10 | Pro-Co | 11 (4-13) | NGS | 67 | 67 | 2017 | rejectiom: ABMR, TCMR, mixed ABMR and TCMR |
| 9 | Verhoeven | 2022 | Netherland | 2018.08-2019.12 | Pro-Co | 59 (N/A) | ddPCR | 223 | 17 | 2019 | ABMR: biopsy-proven acute rejection; histologic evidence of AMR without C4d positivity and without donor-specific antibodies |
| 10 | Zhang | 2020 | China | 2016.11-2017.09 | Pro-Co | 36 (N/A) | NGS | 37 | 37 | 2015 | ABMR: acute active or chronic active ABMR |
| 11 | Jordan | 2018 | United States | 2015.04-2018.08 | Pro-Co | 50 (N/A) | NGS | 87 | 90 | 2013 and 2007 | ABMR：includes only acute/active ABMR and chronic active ABMR |

Prospective cohort (Pro-Co); Retrospective cross-sectional (Ret-Co); next-generation sequencing (NGS); digital droplet polymerase chain reaction (ddPCR); not available (N/A); antibody-mediated rejection (ABMR); T cell–mediated rejection (TCMR); cell–mediated rejection (CMR); Active antibody‐mediated rejection (AMR).

**Table S2 Diagnostic parameters of the literature**

| **Number** | **Reference** | **Year** | **Threshold** | **Rejection type** | **TP** | **FP** | **FN** | **TN** | **AUC** | **Sensitivity(%)** | **Specificity(%)** | **PPV** | **NPV** |
| --- | --- | --- | --- | --- | --- | --- | --- | --- | --- | --- | --- | --- | --- |
| 1 | Bu(1) | 2022 | 0.5 | Rejection | 46 | 46 | 13 | 114 | N/A | 78 | 71 | 50 | 90 |
| 2 | Bu(2) | 2022 | 1 | Rejection | 34 | 29 | 25 | 131 | N/A | 58 | 82 | 54 | 84 |
| 3 | Bu(1) | 2022 | 0.5 | ABMR | 40 | 56 | 11 | 81 | N/A | 79 | 59 | 42 | 88 |
| 4 | Bu(2) | 2022 | 1 | ABMR | 33 | 34 | 18 | 103 | N/A | 65 | 75 | 49 | 85 |
| 5 | Gielis | 2019 | 0.88 | Rejection | 5 | 10 | 8 | 58 | 0.64 | 39 | 85 | 33 | 88 |
| 6 | Whitlam(1) | 2019 | 0.75 | ABMR | 11 | 12 | 2 | 36 | 0.89 | 85 | 75 | 48 | 95 |
| 7 | Whitlam(2) | 2019 | 13cp/ml | ABMR | 11 | 10 | 2 | 38 | N/A | 85 | 79 | 52 | 95 |
| 8 | Sigdel | 2019 | 1 | Rejection | 48 | 45 | 6 | 118 | 0.87 | 89 | 73 | 52 | 95 |
| 9 | Huang(1) | 2019 | 0.74 | Rejection | 27 | 8 | 7 | 21 | 0.71 | 79 | 72 | 77 | 75 |
| 10 | Huang(2) | 2019 | 1 | Rejection | 23 | 8 | 11 | 21 | N/A | 68 | 72 | 74 | 66 |
| 11 | Huang(1) | 2019 | 0.74 | ABMR | 24 | 11 | 0 | 28 | 0.82 | 100 | 72 | 69 | 100 |
| 12 | Huang(2) | 2019 | 1 | ABMR | 20 | 11 | 4 | 28 | N/A | 83 | 72 | 65 | 88 |
| 13 | Bloom(1) | 2017 | 1 | Rejection | 18 | 12 | 12 | 65 | 0.74 | 59 | 85 | 61 | 84 |
| 14 | Bloom(2) | 2017 | 1 | ABMR | 12 | 16 | 3 | 76 | 0.87 | 81 | 83 | 44 | 96 |
| 15 | Oellerich(1) | 2019 | 0.43 | Rejection | 16 | 122 | 6 | 273 | 0.83 | 73 | 69 | 12 | 98 |
| 16 | Oellerich(2) | 2019 | 52cp/mL | Rejection | 16 | 107 | 6 | 288 | 0.73 | 73 | 73 | 13 | 98 |
| 17 | Puliyanda | 2021 | 1 | Rejection | 32 | 0 | 3 | 32 | 1.00 | 86 | 100 | 91 | 91 |
| 18 | Verhoeven | 2022 | 18.7cp/mL | ABMR | 3 | 2 | 1 | 11 | 0.86 | 86 | 80 | 54 | 95 |
| 19 | Zhang | 2020 | 1 | ABMR | 16 | 5 | 2 | 14 | 0.74 | 89 | 74 | 76 | 88 |
| 20 | Jordan | 2018 | 1 | ABMR | 13 | 12 | 3 | 62 | N/A | 81 | 84 | 52 | 95 |

True positive (TP); false positive(FP); false negative (FN); true negative (TN).
